# Research highlights for issue 10: understanding complex lifecycles

**DOI:** 10.1111/eva.12340

**Published:** 2015-11-20

**Authors:** Britt Koskella

**Affiliations:** Evolutionary Applications

Many parasites, including those of relevance to human health, use multiple hosts in order to complete their lifecycle. These complex lifecycles are somewhat mysterious from an evolutionary perspective, as the reliance on more than one host species seems likely to make the parasite more vulnerable to ecosystem perturbation and to restrict its range (López et al. 2015). There have been a number of intriguing hypotheses put forward to explain how selection could favor such strategies – ranging from somewhat neutral explanations, such as selection to simply survive predation of hosts, to elaborately adaptive ones, such as selection to exploit hosts of larger size by moving up the food chain (Parker et al. 2015a; Poulin and Lagrue 2015). However, finding evidence to support these hypotheses – and especially to craft generalizable explanations – has proved difficult.

One way to tease apart these hypotheses is to test the underlying assumptions of selection for expanded host use across systems. For example, what are the consequences of moving up the trophic ladder in terms of lost transmission opportunities or production of propagules? In their recent paper, Robert Poulin and Clément Lagrue aimed to quantify both asexual amplification in intermediate hosts and trophic transmission to definitive hosts for helminth parasites with complex life cycles (Poulin and Lagrue 2015). Looking across parasite species, the authors uncovered a positive correlation between asexual multiplication in the first intermediate host and parasite density at the next life stage for parasites with cercarial transmission (i.e. those with a free-swimming larval stage to move among hosts), but a drop in cohort density across stages for parasites with trophic transmission (i.e. those relying on predation of intermediate hosts). In both cases, however, the authors argue that the expansion occurring during asexual reproduction in the first host more than compensates for lost transmission later in the life cycle suggesting the costs of a complex life cycle might not be as large as expected.

Given the potential advantages of complex life cycles in terms of parasite amplification, the acquisition of host life stages might be predicted to be both adaptive and evolutionary labile. Recent work by Nate Hardy and colleagues utilized comparative phylogenetic analyses to explore the evolutionary flexibility of complex life cycles in aphids (Hardy et al. 2015). By testing for correlations between life cycle complexity and plant host breadth or aphid reproductive mode, the authors discovered a positive relationship between heteroecy (lifecycle complexity) and polyphagy (the ability to eat a variety of food). They also found that life cycle complexity has evolved faster than the Aphidinae speciation rate, a result supporting the potential for rapid response of lifecycle complexity to selection.

The observed evolutionary flexibility of parasitic life cycles raises the possibility that parasites could adapt to multiple hosts simultaneously, using the same mechanism, without trade-offs between growth in one host and growth in another (however, see Parker et al. 2015b for cases where this might not be expected). Indeed, recent comparative work by Daniel Peterson and collaborators on host adaption in plant-feeding insects with pathogen-like life histories suggests that many adaptations allowing increased fitness on one host can also increase fitness on alternative hosts (Peterson et al. 2015). Furthermore, the idea that having an obligate association with multiple host species necessarily limits a parasite’s ability to jump to a new host or invade a new region has also been recently challenged. In their opinion piece, Miriama Malcicka and coauthors use the recent European invasion by the liver fluke, *Fascioloides magna*, a parasite with both an intermediate and final host, as a case study to emphasize the potential for rapid parasite range expansion and host jumps, even in the face of extreme ecophysiological requirements (Malcicka et al. 2015). If generally true, this would suggest parasites with complex life cycles should be more robust to changing abiotic and biotic conditions than might be expected based on their seeming specialization.

Overall, a better understanding of both the selection acting on parasites with complex life cycles and the consequences of these life cycles for adaptation, range expansion, and host switching is critical for predicting the emergence and spread of these often devastating parasites in human, agricultural, and natural populations (Buhnerkempe et al. 2015).

## References

[b1] Buhnerkempe MG, Roberts MG, Dobson AP, Heesterbeek H, Hudson PJ, Lloyd-Smith JO (2015). Eight challenges in modelling disease ecology in multi-host, multi-agent systems. Epidemics.

[b2] Hardy NB, Peterson DA, Dohlen CD (2015). The evolution of life cycle complexity in aphids: ecological optimization, or historical constraint?. Evolution.

[b3] López Z, Cardenas L, Runil F, González MT (2015). Contrasting definitive hosts as determinants of the genetic structure in a parasite with complex life cycle along the south-eastern Pacific. Molecular Ecology.

[b4] Malcicka M, Agosta SJ, Harvey JA (2015). Multi level ecological fitting: indirect life cycles are not a barrier to host switching and invasion. Global Change Biology.

[b5] Parker GA, Ball MA, Chubb JC (2015a). Evolution of complex life cycles in trophically transmitted helminths. I. Host incorporation and trophic ascent. Journal of Evolutionary Biology.

[b6] Parker GA, Ball MA, Chubb JC (2015b). Evolution of complex life cycles in trophically transmitted helminths. II. How do life-history stages adapt to their hosts?. Journal of Evolutionary Biology.

[b7] Peterson DA, Hardy NB, Morse GE, Stocks IC, Okusu A, Normark BB (2015). Phylogenetic analysis reveals positive correlations between adaptations to diverse hosts in a group of pathogen-like herbivores. Evolution.

[b8] Poulin R, Lagrue C (2015). The ups and downs of life: population expansion and bottlenecks of helminth parasites through their complex life cycle. Parasitology.

